# Host parasite communications—Messages from helminths for the immune system: Parasite communication and cell-cell interactions

**DOI:** 10.1016/j.molbiopara.2016.06.003

**Published:** 2016-07

**Authors:** Gillian Coakley, Amy H. Buck, Rick M. Maizels

**Affiliations:** a Institute for Immunology and Infection Research, and Centre for Immunity, Infection and Evolution, School of Biological Sciences, University of Edinburgh, UK; b Wellcome Trust Centre for Molecular Parasitology, Institute of Infection, Immunology and Inflammation, University of Glasgow, 120 University Place, Glasgow, G12 8TA, UK

**Keywords:** Parasite communications

## Abstract

Helminths are metazoan organisms many of which have evolved parasitic life styles dependent on sophisticated manipulation of the host environment. Most notably, they down-regulate host immune responses to ensure their own survival, by exporting a range of immuno-modulatory mediators that interact with host cells and tissues. While a number of secreted immunoregulatory parasite proteins have been defined, new work also points to the release of extracellular vesicles, or exosomes, that interact with and manipulate host gene expression. These recent results are discussed in the overall context of how helminths communicate effectively with the host organism.

## Highlights

• Helminth parasites release a spectrum of mediators to dampen host immunity.
• Secreted proteins can act on host receptors and intracellular signalling.
• Parasites also produce exosome-like extracellular vesicles containing microRNAs.
• Exosomes can enter host cells and modulate host gene expression.
• Extracellular vesicles may be a more general mode of host-parasite interaction.

## Graphical abstract

**Figure:**
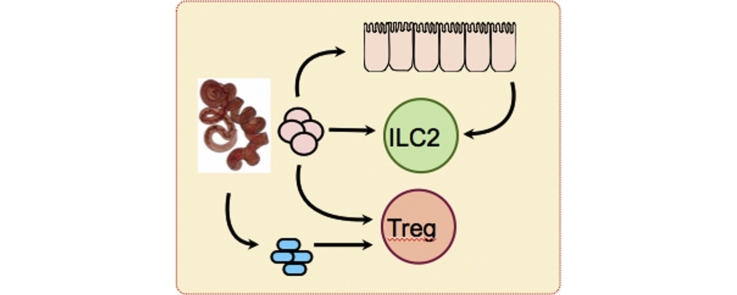

## 1 Introduction

Helminth parasites generally establish long-term infections in their host, reflecting their ability to drive a new physiological and immunological homeostasis that best accomodates the invader [1]. Over eons of evolutionary time, parasites have developed a remarkable suite of finely-tuned molecular adaptations that manipulate, inhibit or activate different host cells or pathways in order to maximise parasite success [2], [3]. In this review, we discuss some of the more recent and exciting developments that shed light on the molecular pathways of host-parasite communication.

Helminths are parasitic worms belonging to the lower invertebrate phyla of nematodes (roundworms) and platyhelminths (flatworms). A wide variety of helminth species are able colonise an extraordinary array of niches and host organisms, in each case circumventing host defence and expulsion mechanisms. Interestingly, the strategy of helminths is not to outpace the immune system through rapid multiplication or antigenic variation, but to manipulate and modulate immunity in order to defuse immune defences, meaning the host fails to eliminate the parasites [4]. Helminths essentially take hold by stealth, first inactivating host detection systems that would otherwise raise the alarm, and then effectively tolerizing the immune system to parasite antigens, and in doing so, also dampening responses to bystander antigens in allergy or autoimmunity [5].

The softly–softly strategy of helminths has implications for how they communicate with their hosts and the immune system of their host, suggesting that there must be a continual dialogue to maintain the state of tolerance. Because infection comprises relatively stable populations of long-lived parasites, it is logical to deduce that the dialogue is conducted by products continuously released from live parasites that address different specific components of the immune system [2]. This notion is supported by observations that most of the immunomodulatory effects of helminth infections are reversed following drug-mediated parasite clearance [6], [7], [8].

Correspondingly, much attention has been paid to the “excretory-secretory” (ES) antigens of helminths, a pragmatic approach to collect mixtures of released proteins that dates back over 60 years [9]. Much more recently, of course, the application of genomics, transcriptomics and mass spectrometry has transformed our understanding of these complex and heterogeneous preparations by defining the individual molecular components that parasites release to modify their environment [2]. As discussed below, these products are not only proteins, but also include glycans, lipids and nucleic acids, in particular miRNAs, as well as small molecules and metabolites, and are released in a variety of “packages”, including lipid vesicles.

## 2 Host recognition of parasites

**Fig. 1:**
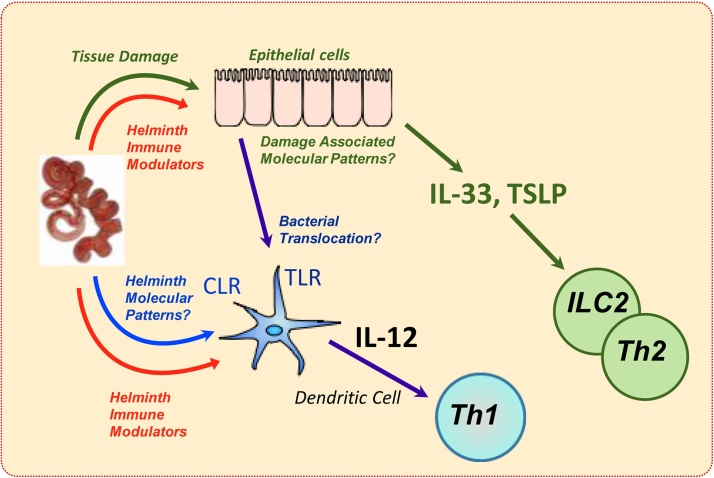
Pathogen recognition systems in helminth infection. Innate mechanisms respond to tissue injury with release of alarmins (eg IL-33, TSLP) which can initiate a type 2 response; helminths can block alarmin release or receptors for alarmins such as ST2 (the IL-33R). Pathogen associated molecular patterns may also be recognised eg by Toll Like Receptors (TLRs) or C-type lectin receptors (CLRs), and these molecular patterns may be directly presented by helminths, or indirectly through bacteria translocating through injured epithelium. In the latter case, the Th1 response driven by IL-12 is blocked by helminth secreted immune modulators.

The first encounter between parasite and host generally entails breaching of a barrier surface (such as skin or intestinal epithelium) that provokes release of ‘alarmins’ [10] and recognition of the invader by pattern recognition receptors (PRRs), such as the Toll-like receptors (TLRs) that drive inflammatory cytokine production. Alarmins, closely associated with helminth-mediated tissue damage, include IL-33 and TSLP [11], [12], which both promote a Type 2 pro-allergic and anti-helminth mode of the immune response. However, helminths can partially or entirely circumvent this threat (Fig. 1); for example, the response of dendritic cells (DCs) to TLR ligation is effectively negated by products from *Nippostrongylus brasiliensis* and other helminths, with IL-12 production being especially inhibited [13], [14], [15], [16], [17] while epithelial cell release of IL-33 is directly blocked by products released by *Heligmosmoides polygyrus* [18]. As discussed in the following section, some of the molecular mediators responsible for blocking innate activation are now being defined.

The archetypal PRRs react to microbial products such as LPS and lipoteichoic acid by triggering production of pro-inflammatory cytokines, such as IL-12, that drive the Th1 response. The consistent ability of varied helminth products to suppress IL-12 release following TLR stimulation may be a mechanism aimed not so much at blocking anti-parasite immunity, but at avoiding collateral inflammation at barrier sites where, for example, bacterial translocation could accompany helminth invasion. Whilst the central role of TLRs in pathogen pattern recognition by the host is now well understood, it is surprising that no parallel recognition system has yet been defined for Th2-inducing organisms such as helminths. However, a few TLR ligands from helminths have been described, including from *Schistosoma mansoni* both the lyso-phosphatidylserine glycolipid [19] and RNA activating TLR3 [20], and other receptor systems such as the C-type lectin receptors (CLRs) may fulfill the role of innate recognition in other settings [21], [22], [23].

## 3 Protein-mediated interactions

The first level of parasite communication with the host can be considered to be simple protein–protein interactions in the extracellular milieu, either with fluid phase host components, or exposed receptors on host cell surfaces. For example, *H. polygyrus* secretes a functional mimic of the immunomodulatory cytokine TGF-β, which ligates mammalian surface receptor and transduces a suppressive signal to T cells (Johnston et al., submitted for publication). Space precludes further discussion of the many individual proteins now found to be involved in host-helminth interactions, but perhaps the most intriguing are members of the CAP superfamily (Pfam00188) which are greatly expanded across all helminth parasite lineages [24], [25], and highly represented in the secreted protein compartments [26], [27]. One member of this family from *Necator americanus* (a hookworm) was one of the first to be characterised functionally as NIF, a secreted inhibitor of integrin binding that blocks neutrophils [28].

While functional assignments for members of the CAP gene family other than NIF are scarce, it is interesting to note that in a plant parasitic nematode, a homologue binds to a tomato plant innate defence protein, disabling resistance pathways and promoting infection [29]. Thus, helminth secreted proteins are not necessarily limited to interactions at the host cell surface, but can perform functions within host cells, raising the question of how they may enter the cell.

### 3.1 Intracellular action of helminth proteins

Two well-studied helminth glycoproteins are known to enter host cells and mediate profound biological effects. The *Schistosoma mansoni* egg-derived glycoprotein ω1 is a ribonuclease bearing Lewis X glycan side chains, that bind to surface lectin of dendritic cells, mediating uptake into the cell, resulting in the protein moiety acting to block protein synthesis [30], [31]. DCs pretreated with ω1 are also switched into the type 2 immune pathway, activating naive T cells to become Th2 effector cells.

A different mediator is the predominant secreted glycoprotein of the filarial nematode *Acanthocheilionema viteae.* This product, ES-62, is a 62-kDa component bearing N-linked phosphorylcholine (PC) sidechains. Through interaction with surface TLR4, ES-62 enters the cell, and in the intracellular milieu the PC moiety interrupts the downstream signalling of both the B cell receptor and TLR4, effectively inhibiting cell activation [32]. A further example is the FheCL1 cysteine protease from *Fasciola hepatica,* which degrades TLR3 in host macrophages thereby inhibiting activation; although TLR3 is an intracellular pathogen sensor, FheCL1 is able to enter the endosome to degrade the receptor in situ [17].

A separate pathway is targetted by the filarial cystatin molecule CPI-2. This protein has two inhibitory sites which target conventional cysteine proteases, and asparaginyl endopeptidase (AEP) respectively [33]. Human B cells exposed to CPI-2 from *Brugia malayi* (a human filarial parasite) are no longer able to process protein antigen for presentation to T cells, a pathway dependent on AEP activity in the endosome [33]. Further studies on a closely related cystatin from *A. viteae*show that this protein is taken up by mouse macrophages and activates ERK and p38 kinases, resulting in the production of immunoregulatory IL-10, in a manner linked to the phosphorylation of the CREB and STAT3 signalling factors [34].

Many other products have been shown to modulate intracellular signalling in host cells, although the mode of entry is not always understood. For example, the ALT-2 protein is derived from an abundant larval transcript of the filarial parasite *B. malayi*. The effect of this protein is seen when added to macrophages, or introduced into the macrophage via transfection of the intracellular protozoan *Leishmania mexicana*, in the induction of the signalling proteins GATA-3 and SOCS-1, which act to induce type 2 responses and dampen IFN-γ dependent inflammatory signals in the cell [35].

### 3.2 Discovery of exosomes

It is now becoming increasingly apparent that extracellular vesicles, and exosomes in particular, play a key role in cellular communication. Exosomes are nanovesicles around 50–100 nm in size that are secreted by virtually all cells to facilitate the transfer of selected cargo, mainly lipids, proteins and RNA species, whilst retaining phenotypic markers from their cell of origin [36], [37]. Exosomes develop within a cell by inward budding of multi-vesicular endosomes, and thus contain components of the parental cell, such as RNAs or proteins, that may be trafficked into the same compartment. The discovery of extracellular vesicles from kinetoplastids, fungi and bacteria drove the theory that exosome-mediated communication could operate on a cross-species platform, whereby parasite-derived exosomes could interact with, and potentially modulate, the host immune system [38]. Only recently have exosomes been recognised as integral products from extracellular organisms like helminths [38], [39].

It has recently been discovered that parasitic helminths produce exosomes. This was initially reported in the excretory-secretory components of the trematodes, *Echinostoma caproni* and *Fasciola hepatica*, which infect the gastrointestinal tract and liver respectively [40], and in the nematode *H. polygyrus,* which infects the small intestine [41]. Data from the trematode studies further suggests that ES-derived exosomes are capable of reaching the host environment, as they appear to be found intact on the parasites’ tegument. Further support of this is demonstrated by the internalisation of helminth exosomes by host intestinal epithelial cells, suggesting their capacity for cross-phylum communication between helminths and mammals.

The formation of exosomes by helminths had originally been established in free-living nematodes, with the demonstration that *Caenorhabditis elegans* use a novel secretion pathway from the apical membrane, to co-secrete multivesicular bodies, containing exosome-like vesicles, with peptides that normally promote cuticle development. [42]. Exosomes from helminths and protozoa appear to share many specific markers, with those known to be present in mammalian exosomes, such as Heat-shock protein 70 (HSP70), endosomal sorting components e.g. Alix, and surface tetraspanins including CD9 and CD63 [37]. For example, it was shown that whilst exosomes secreted by *Leishmania*-infected macrophages undergo a series of phenotypic changes following infection, they still retain some typical exosome markers, including TsG101, Alix and CD63 [43]. Additionally, transcriptomic analysis of the cestode, *Echinococcus granulosus,* revealed the existence of other CD63-like tetraspanin family members [44]. Tetraspanins were independently selected as target candidates for vaccination against *Echinococcus multilocularis,* another tapeworm which causes alveolar echinococcosis, a highly fatal disease dominating parts of Siberia, Central Europe and China [45], [46]. The focus on a tetraspanin-targeting vaccine is also being explored against the human pathogen *S. mansoni* [47], [48].

**Fig. 2:**
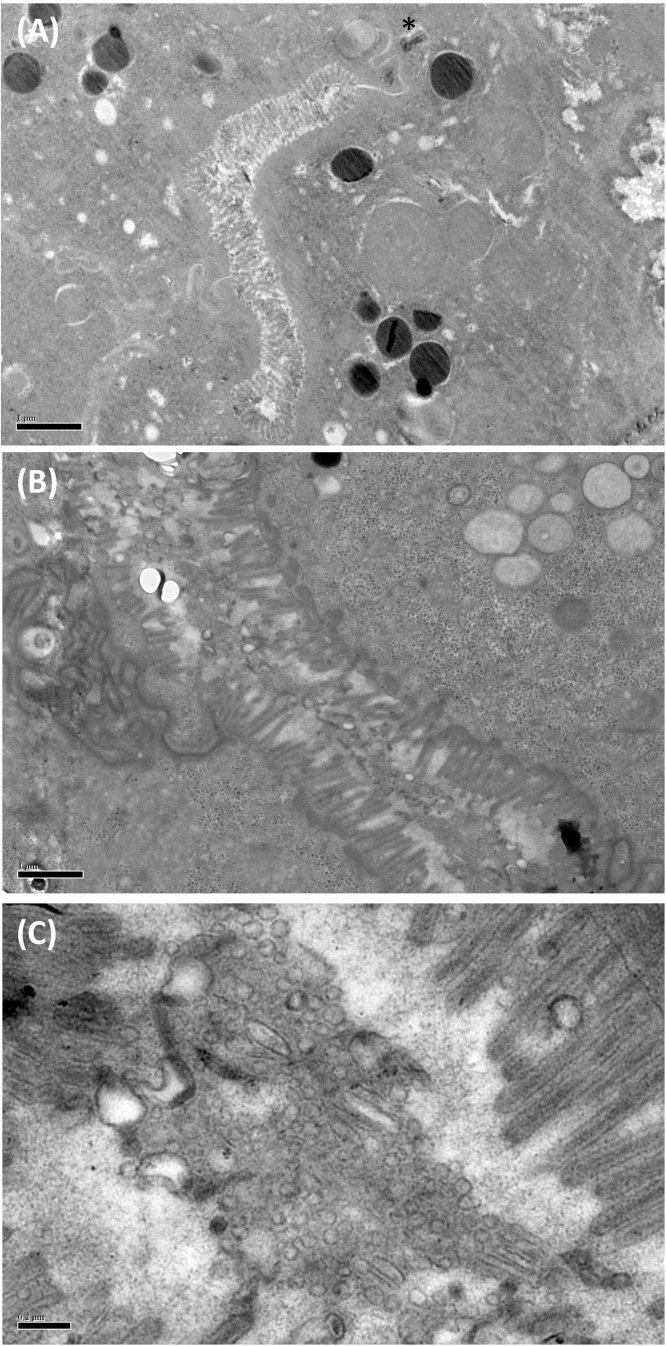
Proposed route of secretion of exosomes by the nematode *H.polygyrus*. (**A**) Low-power micrograph of the intestinal tract of adult *H. polygyrus* showing brush border epithelium, as well as ducted secretory gland (marked with asterisk). (**B**) Higher power image *of H.polygyrus* intestinal ultrastructure, with (**C**) zoom of the luminal contents containing a large number of vesicles and macromolecular structures consistent with the presence of exosomes.

Previously, we have shown the ability of *H. polygyrus*, a murine gastrointestinal nematode, to secrete exosomes that contain multiple miRNA species, as well as a significant number of proteins, representing approximately 10% of the total protein secretion of an adult worm [41]. Proteomic comparison of the secreted products represented in the soluble and vesicular fractions separate by ultacentrifugation also demonstrated enrichment of a number of key components within the exosomes. Interestingly, some of these were proteins which have previously been located at the apical membrane of intestinal epithelial cells of *C. elegans*; electron microscopy also recorded multi-vesicular bodies in the intestinal tissues of *H. polygyrus* adults and exosome-like structures released into the lumen [41], strongly suggesting that the parasite releases exosomes from its alimentary tract (Fig. 2).

**Fig. 3:**
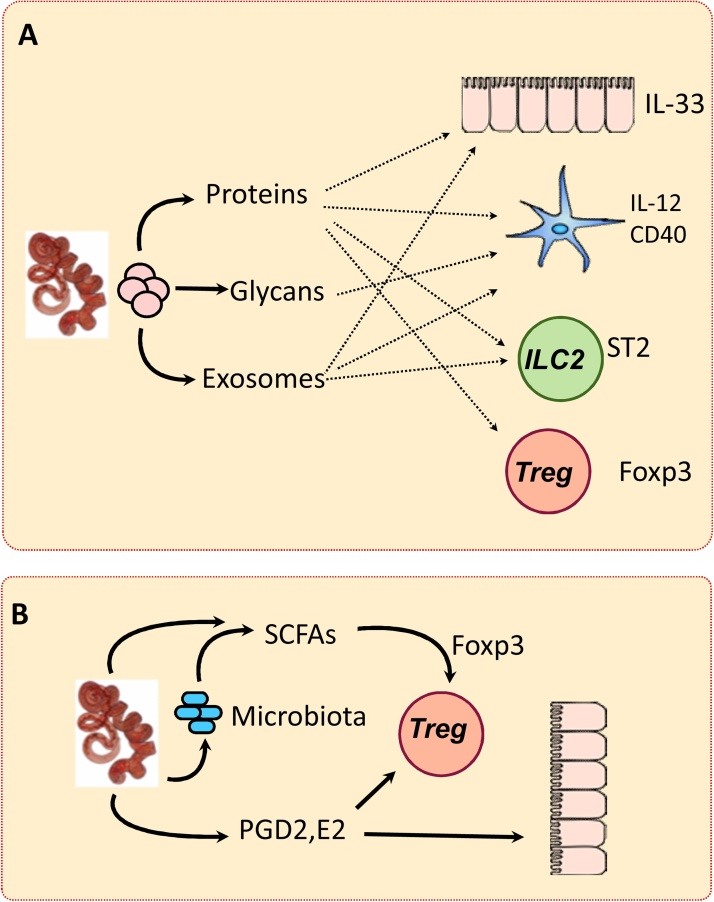
Helminths release diverse molecular species to communicate with host cells, including proteins, glycans and exosome components, including miRNAs (**A**). They also produce short chain fatty acids (SCFAs) and promote commensal microbes which release SCFAs, expanding the Foxp3+ Treg population (**B**).

Functionally, we were also able to show the immunomodulatory capacity of exosomes derived from extracellular helminths. When given prophylactically, *H. polygyrus* exosomes suppress the innate immune response to the fungus *Alternaria alternata*, commonly associated with respiratory allergies, primarily through the modulation of type 2 innate lymphoid cells (ILC2s) [41]. Activation of ILC2s normally drives eosinophilia through the release of IL-5, which is blocked by parasite exosomes (Fig. 3). Moreover, *H.polygyrus* exosomes have been shown to reduce the expression of IL1RL1/ST2 transcript, both *in vitro* and *in vivo* in murine cell populations. This gene encodes the IL–33 receptor,and is required for the type 2 immune response to be initiated by ILC2s, consistent with the observed protection from allergic inflammation conferred by exosomes *in vivo*. The importance of the IL-33 ligand-receptor axis in anti-parasite responses has also been well-documented [18], [49]. Thus, our data demonstrated the ability of *H. polygyrus-*derived exosomes to avoid parasite clearance by modulating this key aspect of the host immune response.

Parallel studies on the digenean trematode cattle parasite, *Dicrocoelium dendriticum*, also found exosomes to be released into culture medium, and to contain over 80 protein components as well as at least 30 miRNA species with identity or near-identity to known sequences [50]. Although no functional tests were reported, the authors highlighted the commonality with the major Schistosome miRNAs Bantam, miR-10 and miR-3479 that are detectable biomarkers in the plasma of infected hosts [51].

Most recently, Nowacki et al. described 30–100 nm exosome-like vesicles secreted by *S. mansoni* schistosomulas that are enriched in specific non-coding RNAs and proteins [52]; over 200 miRNAs were identified as well as 20 tRNA-derived small RNAs and over 100 proteins. In addition, it was shown that the L3 infective stage of *B. malayi* secrete 50–120 nm vesicles rich in miRNA species, and a protein complement that included not only classical exosome-associated products, but those with potential to interfere with host cell responses, such as Cathepsin L [53]. Significantly, the infective stage was found to be much more prolific exosome producers than the adult worm stage, possibly reflecting the demands of transition from vector to host at this point in the life cycle. Sotillo et al. further reported that adult *S. mansoni* worms release 50–130nm-sized exosome vesicles, containing over 80 identifiable proteins 5 of which are tetraspanins and an abundant saposin-like protein [54]. These authors also highlighted that a number of known Schistosome vaccine candidate antigens, including the tetraspanins discussed above, are prominent components of the exosomes. In the related parasite, *S. japonicum*, Wang et al. reported that 30–100 nm vesicles released by adult worms cultured in vitro for 5 h, detectable upon ultracentrifugation of the culture medium [55]. These authors also found that *S. japonicum* exosomes stimulated the murine macrophage-like cell line RAW264.7 to produce nitric oxide alongside other indicators of a Type 1 pathway, although in this study the protein cargo of the exosomes was not identified. The presence of many key proteins, as well as RNA species, in the secreted vesicles highlights both the complexity and diversity of cargo within exosomes, with a correspondingly wide range of potential interactions within recipient cells [56].

A broader scope for helminth exosomes has also emerged from analyses of the liver fluke *Opisthorchis viverrini*, a trematode prevalent in parts of South-East Asia where it is causally linked to cholangiocarcionoma (bile duct cancer). As with the species described above, secretory material contained exosomes (measuring in this case 40–180 nm), with a similar spectrum of associated proteins including tetraspanins [57]. Some exosome-associated proteins were also found in the bile fluid of infected hosts. Exosome entry into host cells was blocked with anti-tetraspanin antibody, arguing that this protein is likely to be exposed on the vesicular surface as found for mammalian exosomes. Most significantly, *O. viverrini* exosomes were found to stimulate cell proliferation in a human cholangiocyte cell line, and to also induce their production of the pro-inflammatory cytokine IL-6 in a manner that was partly inhibitable by anti-tetraspanin antibody. Taken together, these data make a strong case that *O. viverrini* drives potentially tumorogenic changes in the host bile duct that could account for the carconogenic effects of infection with this parasite.

### 3.3 Helminth miRNAs in exosomes

It has been well documented that non-coding RNAs, and microRNAs in particular, transfer between cells and organisms through their encapsulation within exosomes and other extracellular vesicles [58]. Indeed, this provides a mechanism for protecting RNAs from degradation when outside of the cell, and presumably enables an uptake pathway to deliver RNA to the appropriate cellular compartment in the recipient. Several of the studies discussed above identified small RNAs within parasite-derived exosomes, including those from the nematodes *B. malayi* [53] and *H. polygyrus* [41], and the trematodes *D*. *dendriticum* [50] *and S. mansoni* [52].

In the case of *H. polygyrus,* we were able to show a suite of RNA species packaged within exosomes, including miRNAs such as let-7, miR200 and bantam [41], which could suppress the mouse phosphatase Dusp1 using a reporter assay. New data identifying extensive miRNA repertoires in parasitic helminths are now becoming available, although the distribution of these within secretory exosomes has in most cases yet to be established.

Most importantly, definitive evidence for helminth-derived miRNAs acting on host genes remains to be obtained; however, the circumstantial evidence remains enticing; not only are extensive seed sequences shared between helminth and host miRNAs, but the miRNA-rich exosomes (of *H. polygyrus* at least) also carry the worm Argonaut protein [41], [59], suggesting that a functional package for gene repression is being delivered to the target cells.

## 4 Small molecule interactions

Increasing attention is being paid to how small molecules, metabolites, hormones and molecular cues, are intimately involved in intercellular communication. For example, the short-chain fatty acids (SCFAs, butyrate, acetate and proprionate) are not produced by the mammalian organism, but are derived from commensals at levels that promote regulatory T cells [60]; hence dysbiosis can be pathogenic due to disruption of this pathway [61], [62]. Interestingly, helminths can also synthesise these compounds, [63] as well as promote the commensal bacteria able to produce significant quantities of SCFAs [64].

Other small molecules include prostaglandins D2 and E2 produced by filarial parasites *B. malayi* [65] and *Onchocerca volvulus* [66], and by the skin-invasive cercariae of *S. mansoni* [67]. In addition to small molecules and metabolites, helminths also directly modify host-derived small ligands such as acetylcholine (through acetylcholinesterase [68]), platelet-activating factor (PAF hydrolase [69]) and ATP (apyrase [70]), among many others, a discussion of which are beyond the scope of the current review.

## 5 Interactions through the microbiome

Helminth parasites, particularly in the intestinal tract, share their niche with a myriad of micro-organisms, principally hundreds of bacterial species known as the microbiota [71], [72], [73]. Notably, helminth infections depend to a great extent on the presence of these commensals: for example, in the absence of caecal bacteria, *Trichuris* eggs do not hatch in the intestine [74]. Most studies of the microbiota in mice infected with intestinal helminths have found significant and occasionally sweeping changes in the species composition, particularly among *Bacteriodes* and *Lactobacillus* populations [71], [72], [73]. Recently, it was found that BALB/c mice infected with *H. polygyrus* showed expansion of the *L. taiwanensis* species, and that the degree of colonisation with this bacterium positively correlated with both adult worm numbers and the level of Treg activation [75]. Interestingly, if mice were given *L. taiwanensis* prior to receiving *H. polygyrus* larvae, they were rendered more susceptible to infection, establishing a mutual promotion between the bacteria and helminth organisms.

It has also been suggested that the immune modulatory effects of helminth infection may be mediated in part indirectly, through altering the intestinal microbiome. To date, intriguing experiments have been reported in which the intestinal contents of infected mice (containing bacteria but also a range of host and parasite products) are able to dampen the allergic response when transferred to recipient mice [64]. It will be fascinating to analyse this effect in more detail, particularly if as with *L. taiwanensis*, an individual bacteria is found responsible.

Significantly, a recent report has shown that faecal miRNAs derived from intestinal epithelial cells can influence the microbiome, potentially through direct interactions with bacterial genes [76]. These miRNAs were suggested to be present in extracellular vesicles, raising the possibility that both host and helminths could modulate the microbiome through this novel mechanism, and indeed as mentioned below, that host exosomes could impact on the helminth organisms parasitising the intestinal tract.

## 6 Two way street − helminths listening to their host

While the focus of this review has been on how helminths deliver messages to the host immune system, there are some intriguing examples of how helminths also detect and respond to host immune status. Classic studies on *N. brasiliensis* found that the adult worms adapt to an immunised host by switching expression levels and isoforms of secreted acetylcholinesterase [77]. More recently, detection of host cytokines has been found in Schistosomes, which require the presence of host TNF to mature to egg laying [78] and filarial parasites responding to high IL-5 levels *in vivo* by accelerating their maturation and production of offspring [79]. An example of a helminth receptor able to ligate a host cytokine was established in the case of the TGF-β family receptor of *S. mansoni* [80].

An intriguing possibility that extracellular vesicles from the host provide a channel of communications that influence the helminth parasites, although as yet there are no reports of parasites being directly receptive to vesicle-mediated signals. However there is a growing literature demonstrating the use of host-derived extracellular vesicle impact on defence against pathogens. For example, exosomes derived from IFN-α stimulated cells were able to induce antiviral activity and limit viral replication in recipient infected cells [81], [82]. Furthermore, human semen exosomes have also been implicated in resistance to HIV-1 following their uptake into naïve cells by reducing viral fitness [83]. Another example of exosome-mediated host defence is demonstrated during the innate response to protozoan parasite *Cryptosporidium parvum.* TLR4-mediated activation of the host epithelium induces the release of antimicrobial peptide-containing exosomes that limit infectivity of the pathogen in the intestinal environment [84]. The development of a directed anti-pathogen response by host exosomes has also been explored for use in a more clinical setting, in which host exosomes collected from parasite antigen-primed dendritic cells induce protection from different protozoan infections, including *Toxoplasma gondii* [85] and *Leishmania major* [86].

## 7 Conclusions and outlook

Helminths have accompanied a vast range of host species throughout evolution, developing sophisticated pathways of communication with, and even control of, the immune system of their hosts. The rapid discovery that many helminth species have the ability to release exosomes to mediate cross-phylum interactions speaks to the importance of this pathway in host-parasite biology. In this new light, we now see how the large extracellular parasites, classified as helminths, may be able to “reach in’ to the intracellular machinery of host cells, modifying their behaviour in ever more remarkable ways. As exosome uptake is not necessarily receptor-dependent, it is difficult for the host to evolve counter-measures to block parasite exosome effects, while it would be relatively easy for the parasite to exploit exosome traffic for effective interference molecules, from proteins and enzymes to small RNAs and other modifiers of gene expression. Furthermore, these vesicles offer a robust vehicle for parasites that may have to deliver their ‘message’ through extracellular spaces of very different nature, and quite possibly through cells and tissues too.

Greater understanding of helminth exosomes, however, should direct us to ways of neutralising their effects, building on our existing knowledge of immunomodulatory proteins and glycans. For example, exosomes may constitute good vaccine targets, if we can generate antibody responses to key surface membrane components that are required for cell entry. In addition, new drug targets may emerge from defining the pathways required for exosome biogenesis in helminths, and/or the events within the host cell that follow helminth exosome uptake. Hence, a new window has opened not only on how helminths defeat the immune system, but on how we can turn the tables and defeat the strategy of the helminth.
